# The Hospital. Nursing Section

**Published:** 1904-10-01

**Authors:** 


### The Hospital.

### Nursing Section.

Contributions for this Section of "THE HOSPITAL" should be addressed to the EDITOR, "THE HOSPITAL," NURSING SECTION, 28 & 29 Southampton Street, Strand, London, W.C.

No. 940.—VOL. XXXVII. *SATURDAY, OCTOBER* 1, 1904.

### Notes on News from the Nursing World.

### OUR CHRISTMAS DISTRIBUTION.

WE are glad to be able to announce this week that we have already received contributions to our Christmas distribution of articles of clothing for the use of patients in hospitals and infirmaries. They consist of a parcel of flannelette shirts from No. 37 Royal National Pension Fund, and a baby's knitted coat. All parcels should be addressed to the Editor, 28 and 29 Southampton Street, Strand, London, W.C., with "Clothing Distribution" written on the outside.

### LONDON NURSES AND PROFESSOR FINSEN.

ALTHOUGH all nurses know and honour the name of Finsen, few of the many thousands in England know the celebrated Danish scientist personally. That pleasure however belongs to the two ladies who, at the time that the first lamp for the cure of lupus was introduced into the London Hospital at the instance of the present Queen, were sent out to receive his personal instructions and to see the light at work. Although neither the assistant matron nor her companion was conversant with the Danish language, as one of them was fairly proficient in German, she found it quite possible to converse without difficulty, nearly all Danish doctors being able to talk German more or less fluently. Professor Finsen himself was extremely kind to the two Englishwomen, and he gratified them before they left Copenhagen by presenting them with his signed photograph, which now hangs in the sisters' room attached to the light department at the hospital. Even as long ago as 1899 Finsen was almost an invalid, but the two nurses who noted his cheery manner, his frank, open expression, and his bright smile felt it difficult to believe that he who was spending his life trying to save others from suffering was, as he himself knew, a doomed man.

### STREET COLLECTIONS BY HOSPITAL NURSES.

IT is satisfactory that the amount contributed in Salisbury Cathedral at the offertory in aid of the infirmary, after an excellent sermon by the Dean, was higher than it has been since 1897. But we regret that in connection with the anniversary service of the Salisbury Infirmary a number of nu·ses were employed to make collections in the streets. It is true that the sum obtained by this method was £10 5s. 5d., but our opinion is that the end does not justify the means. We object, as a matter of principle, to nurses in uniform soliciting money in the public thoroughfares for any cause whatever, and we trust that the innovation which has been unfortunately introduced under the shadow of one of our great cathedrals, will not become a custom. Street collections, under almost any circumstances, are open to criticism, but when hospital nurses in uniform are pressed into the service because it is thought that they will prove irresistible to persons who would otherwise refuse to give, it becomes necessary to enter a protest. Their duty is to attend the sick, not to run about the streets.

### NURSING IN THE AUSTRALIAN BUSH.

ENGLISH private nurses who are in the habit of grumbling if the friends of their patients do not provide every convenience for them, may think their lot less hard when they read the experiences of nursing in the Australian Bush which we publish to-day. It is true that the circumstances were exceptional, even in Australia, and that in this country they would be impossible. But in the pursuit of their avocation across the seas, nurses must be prepared for the unexpected, including a combination of hard work, rough fare, a lack of necessaries, and an abundance of practical difficulties which can only be overcome by the exercise of much patience, tact, and resourcefulness.

### THE HOSPITAL FOR CONSUMPTIVES AT NORTHWOOD.

SINCE July the matron and sisters of the new consumption hospital at Northwood have been working hard to get it in order, and last week, without any ceremony, this institution was opened. The nursing staff consists of a matron (formerly senior sister at the Mount Vernon Hospital), two sisters, two staff nurses, one of whom will act as night charge-nurse, and four probationers. The nurses' home at Northwood is particularly comfortable, and each nurse has a separate bedroom. Nurses will be able to play tennis and croquet in the grounds. The hospital curriculum will be much the same as that of the Mount Vernon Hospital at Hampstead, of which it is a branch. Probationers in their first year of training will receive a course of lectures from the matron on elementary anatomy and physiology, and nursing. She will also give a course of bandaging lessons. In their second year lectures on advanced anatomy and physiology will be given by the resident medical officer, and there will be a four months' course of instruction in theoretical and practical dispensing. A certificate will be awarded on the completion of two years' training if the final examination is successfully passed. The regulations are similar to those of Mount Vernon Hospital, although the nursing staff at Northwood will be in every way separate.

### THE CONDITION OF THE PARIS PROBATIONERS.

The new nurses' school attached to the Salpêtrière Hospital in Paris is now rapidly approaching completion, and will be an important departure in the right direction on the part of the municipal authorities in Paris. To the Salpêtrière belongs the honour of making the first attempt at giving some systematic training to women anxious to devote their energies to tending the sick and suffering. The attempt was made in 1878, and the example was followed a few weeks later by the Bicêtre. Later, in 1884, M. Bourneville—to whom the advocates of trained nursing in France owe so much—succeeded in getting a regular course of training established at the hospital à la Pitié, whilst as recently as this year a fourth school was created at Lariboisière. But this is the first time that a successful endeavour has been initiated to improve the condition of the Parisian probationer. The nurses who will be trained in the school will not, as heretofore, be required to herd together in damp, cold, ill-ventilated attics, but separate bedrooms will now be provided with all reasonable comforts. It is hoped that these better conditions will attract a class of nurses superior to that which has hitherto attended upon the sick poor in the Paris hospitals.

### AMATEUR NURSES AND WORKHOUSE INFIRMARIES.

The Pontypridd guardians have just refused to entertain a request on the part of the wife of a local Nonconformist minister to enter the workhouse infirmary in order "to gain a knowledge of nursing," with the view of utilising it among the members of her husband's congregation. The guardians are of opinion that the admission of amateur nurses might lead to friction amongst the nursing staff. It would also, we think, be very detrimental to the interests of the patients. There are plenty of training schools open to ladies who wish "to gain a knowledge of nursing," but there would be no advantage in getting rid of pauper assistants in workhouses if amateur nurses were to be allowed to take their place.

### THE NEW HOSPITALS AT GLASGOW.

The nurses' homes in connection with the new hospitals which have just been opened at Glasgow, under the auspices of the Glasgow Parish Council, are notable. At the Stobhill General Hospital, with beds for 1,650 patients, there is a home which provides for the accommodation of a hundred nurses; at Duke Street Hospital, with 244 beds, there are quarters for thirty; and at Oakbank Hospital, with 207 beds, the home holds thirty-five nurses. The buildings at Stobhill are for the infirm class of poor, and for children; and the other two are reserved for acute cases.

### UNIFORMS IN THE NETHERLANDS.

It is notable that none of the nurses in the hospitals in Holland wear caps. Their pretty cotton dresses, white aprons, collars and cuffs are neat and spotlessly clean. The fact that they are bareheaded is emphasised by the fascinating white caps of the Dutch wardmaids. A peculiarity of the district nurses in the Netherlands is that when they are out on their rounds, even while bicycling, they wear their aprons without a cloak, at any rate in summer. Their costume is simply indoor uniform, with the addition of a regulation bonnet and a long veil hanging at the back.

### DISPENSING FOR PROBATIONERS.

Probationers at the Mount Vernon Consumption Hospital, Hampstead, are fortunate in having a four months' course of practical and theoretical dispensing arranged for them during their second year's training. This particular feature of the curriculum was introduced by the present matron, who found that the second year's training in a special hospital often drags. And even though a nurse may never actually have to dispense, an accurate knowledge of all poison doses, and incompatibles, cannot fail to be of lasting benefit to her—and also to her patients.

### BRITISH GYNÆCOLOGICAL SOCIETY.

At the September examination of the British Gynæcological Society the following candidates were successful in obtaining certificate for gynæcological nursing:—Miss E. M. Halliwell, Matron of the Samaritan Hospital for Women, Liverpool; Miss Eveline Marcon, St. Bartholomew's Hospital, London; Miss Etty Moorhouse, South Devon Hospital, and Jessop Hospital for Women, Sheffield; Miss Kitty Read, Grimsby Hospital, and Hospital for Women, Brighton; Miss Sarah Radford, Miss Kate Sanderson, and Miss Lucy Scott, Bagthorpe Infirmary, Nottingham. The written paper was answered by the candidates in London, Nottingham, Grimsby and Whitehaven, under supervision, on September 15th; the *viva voce* examinations were held in London on the 22nd. The candidates were required to describe fully how they would make and apply glycerine plugs; exactly how they would pass the catheter after an abdominal section, and an operation for ruptured perineum; how they would prepare a patient for amputation of the breast, and what subsequent nursing [illegible] would require; how they would prepare the instruments and the dressings for a case of abdominal section; to give a brief report of some gynæcological case which they had nursed; and to describe fully the usual dietary for a patient, for the first week after abdominal section had been performed.

### THE CALIFORNIA STATE NURSES' ASSOCIATION.

We cannot congratulate the members of the California State Nurses' Association upon the first number of what is apparently their official organ. The shape and the appearance are not attractive, while the personal items of the periodical are scarcely in good taste. It, however, contains an interesting article on Visiting Nurses in San Francisco. They have recently been appointed by the Board of Health auxiliary health inspectors, and have been given badges, and papers to serve on those who fail to obey the instructions of the nurse inspectors. So far they have used their authority chiefly in enforcing the law that rubbish shall not be left in yards, or in the streets, or in vacant lots. Another contribution is by a nurse at a quarantine station, who mentions that on boarding the Oriental steamers the nurses have sometimes taken as many as 150 to 200 temperatures.

### BRAVERY OF A NURSE.

At Thrapston, on Monday, a nurse was walking with a patient who had been out of health for some time by the banks of the little river Nene, when the patient, the wife of a well-known tradesman, suddenly ran away from her and jumped into deep water. Without hesitation the nurse rushed after her, and, at the risk of her own life, attempted to rescue the unfortunate woman. She did not succeed, and, no one being near, she then ran to the town for assistance. By the time she was able to obtain it and the body was recovered, a quarter of an hour had elapsed, and efforts to restore consciousness under the superintendence of a medical man proved futile.

### THE MIDWIVES ACT.

The Council of the Incorporated Medical Practitioners' Association have passed a resolution protesting against the action of the Central Midwives Board in deciding to appoint as examiners for the certificate of the Board non-medical women, which, they affirm, raises to the level of the duly registered medical practitioners who have undergone an expensive course of study and a searching examination "women who are not required to show proof that they possess any knowledge of the different subjects which a medical practitioner has to study in order to obtain a diploma." We do not think that the resolution of the Incorporated Medical Practitioners' Association reflects the views of the medical profession.

### EUCHRE AND BENEVOLENCE.

An unusual method has been adopted in Australia for the purpose of assisting the Royal Victorian Trained Nurses' Association Benevolent Fund, in the shape of what is called a Progressive Euchre Party. Prizes were offered consisting of a gentleman's silver-mounted umbrella, and two purses and a silver button hook, both of which went to ladies. The result of the contest, it is stated, was an addition of £30 to the Benevolent Fund.

### COOKERY EXAMINATIONS AT CAMBERWELL.

At Croydon Union Infirmary, as we reported last week, it has just been decided to issue cookery certificates to probationers who pass the necessary examination. There have recently been held at Camberwell Union Infirmary both practical and theoretical examinations in sick cookery. Sixteen nurses were examined in each class. At the practical examination 14 gained first-class and 2 second-class marks, and at the theoretical examination 2 first-class and 11 second-class marks. Certificates have since been presented by the Infirmary Committee to the successful nurses.

### CHIROPODY AND NURSING.

It was announced at the annual meeting of the Sheffield Nurses' Home and Training Institution that the engagement during the year of a specially-trained chiropodist and masseuse to attend persons either at their homes or at the institution had proved a success, and that the demand for her services was steadily increasing. The staff now consists of 25 nurses, and the accounts show that their earnings last year amounted to £311, a rather small total. Votes of thanks were passed to Drs. Keeling and Wilkinson for their gratuitous attendance on the nurses.

### UNFAIR TO NURSES.

There is really no excuse for the *Daily News*, which on Monday headed a report of a case in one of the police courts "Thefts by a Nurse." Even in the report of our contemporary, it was stated that the woman, who was sentenced to four months' hard labour, had falsely represented herself to be a nurse belonging to the London Hospital. We hope as a matter of justice to nurses that on future occasions the *Daily News* will use a correct headline, and not assume that because a person wears in the dock the attire of a nurse she is entitled to be described as one.

### MORE NIGHT NURSES WANTED AT WOLVERHAMPTON.

The Wolverhampton Guardians will doubtless act upon the recommendation of their Nursing Committee, who have reported that the workhouse nursing staff is inadequate. They agree with the medical staff, who have strongly urged the absolute necessity of increasing the number of nurses for night duty, that each ward should be placed in charge of a probationer at night. Seven more probationers will then be required, and in that case both the medical staff and the Nursing Committee are of opinion that one night charge nurse instead of two will be sufficient. By the adoption of this proposal the annual cost of temporary nurses would be diminished.

### AN APPOINTMENT IN BRITISH CENTRAL AFRICA.

We understand that Miss S. A. Claridge has been appointed matron of the Shirè Highland Railway Hospital, Chiromo. The choice is notable inasmuch as Miss Claridge is the first trained nurse who has been given an appointment in that part of British Central Africa. She was trained at the General Hospital, Birmingham. For some time she was attached to the staff of the Staffordshire Institute for Nurses, and as a member of the Army Nursing Service Reserve she subsequently served in South Africa. She has also been sister at the Beira and Mashonaland Railway Hospital, Beira, Portuguese East Coast, Africa.

### TWEEDLEDUM AND TWEEDLEDEE AS NURSES.

A short time ago, in a large provincial children's hospital, a baby developed an attack of measles. As none of the hospital nurses could well be spared for the special isolation necessary, it was decided to engage two private nurses from an outside institution. A porter, new to his work, was told to telephone for the two nurses to be sent at once. In due course two tall and solemn-looking broad men, dressed exactly alike, and each carrying a small black bag, presented themselves at the hospital saying that they had come in answer to the telephone summons to nurse the special case! When they heard that it was a tiny baby they were dumbfounded. However, the matter was soon explained; the new porter had used a wrong number by mistake, and telephoned to a male nurses' co-operation. The two big men, looking exactly like Tweedledum and Tweedledee, were then to their great relief, allowed to depart; and a fresh telephone message soon brought two regulation, befrocked and becapped nurses for the little measles baby.

### The Nursing Outlook.

"From magnanimity, all fear above;
From nobler recompense, above applause,
Which owes to man's short outlook all its charm."

### CLASS LEGISLATION.

An important point in the evidence given in favour of registration of nurses was brought out in an article in the *Daily News* of September 21st. The writer is in favour of some form of register, but says:—

The fatal and cruel blot upon the proposals fathered by Dr. Fenwick and others is that these proposals set up a standard which, at one fell swoop, will cast the nurses of the poor into the outer darkness of inefficiency. "Do you limit this question to the richer classes?" asked Sir John Stirling Maxwell, and Dr. Fenwick replied: "This private nursing entirely affects the richer classes. They do not know who they get into their houses, and they are the people who suffer. They have no supervision over the nurse, day or night, so it is absolutely essential that she should be well trained. They have no guarantee at present that she has been."

And further on he quotes the remark of Miss Isla Stewart that she would keep cottage nurses "outside the scheme." He might have added the evidence of Miss Huxley, who said that they were "not nurses," and of Miss Hughes, who said "only a quarter" of the present nurses would come upon the register.

It is strange that the mass of nurses have not grasped that it is proposed to have a form of legislation which will grant privileges to one quarter of them, and that the other three-quarters are to be left in "outer darkness." It is not only the cottage and district nurses, but those who have trained in small hospitals or in infirmaries which are not recognised as training schools; in fact a stigma will be cast on some of the most devoted and the most worthy workers in our ranks if the proposals of those who have given evidence so far in favour of registration are accepted.

Let there be no mistake about the matter, things have been smoothed over at meetings, and vague promises made in letters, but for one shilling can be had through any bookseller the official report, and in the committee room the fact that class legislation is desired is made abundantly evident.[1] For instance, Miss Hobbs in giving evidence on behalf of the Royal British Nurses' Association which has drafted one of the Bills before the House, in reply to Mr. Hobhouse, said she had no experience outside London and that her experience on the question of nursing the poorer classes was not sufficient to enable her to give an opinion, and that she had no knowledge of the fact that the existence of the registered nurse would prejudice in the minds of the public the status of the unregistered nurse. "My point" said Mr. Hobhouse, "is that there is a large public outside London —outside the class of persons you nurse—whose needs have to be considered, and that you are nursing a very small class of the public indeed."

Now it is not only that there is a large public outside London who need nursing, but also that there are numbers of excellent nurses outside London, who most assuredly do not want to be made "outsiders" by a form of class legislation. And if the country nurse does not want the form of registration now proposed, most certainly the London nurse does not, as has been made abundantly evident to Miss Dock at last. In the September number of the American Journal of Nursing she writes:—

It must be frankly admitted that of the great London hospitals—nine in all, if I recollect rightly—only one, St. Bartholomew's, is willing to recognise us. Only Miss Isla Stewart, of all the London matrons, is willing to affiliate with us. St. Thomas's Hospital, where Miss Nightingale established the first training-school, and which we might say was the mother of us all, stands aloof and regards us and our ways with cold disapproval. Miss Nightingale herself disapproves of State registration, holding that nurses should remain in the control of their training-schools. In Germany, similarly, the great nursing institutions of Berlin and Hamburg will have nothing to do with us, and Kaiserswerth, our grandmother, so to speak, which we all revere, would simply not believe it possible that women emancipated from their hospital authorities could be good nurses.

All this is most regrettable, for in these historic hospitals are women whom we would be glad and proud to know. And, perhaps, they might accept us individually, but we could not get far with them, for they are not in sympathy with much that we are doing in our organisations.

The London matrons, with the sole exception of Miss Isla Stewart, are opposed to State examination and registration.

And Miss Stewart is only the follower of the adventurers who are really running registration Is it not absurd then that the time of the House of Commons should be taken up by these faulty schemes?

Miss Dock is incorrect, however, in stating practically all the London matrons are opposed to State or a Central Examination Board. Far from this being so, at the present time, very quietly, a real Nursing Council is being formed which is truly representative, and which will be able to aid and help nurses as a whole, and not only one class of them. A Council which knows the needs of both country and town, of rich and poor, of nurse and patient, will be able to do a great deal to consolidate and advance the nursing cause, without at present seeking the aid of Parliament. We must have more unity amongst ourselves, more conformity in training, before we are fit to rule ourselves; and most certainly we do not intend to be ruled by outsiders. The Princess Christian spoke very strongly once about rendering justice to the elderly nurses who had not had modern advantages; and we have in these pages constantly pleaded that no slur should be cast on the mental nurses or the infirmary nurses. In fact it is obvious that class legislation is not wanted, and once the trend of the present bills is made plain, the whole wave of public opinion will be against them, and they will vanish away.

[1] Report of the Select Committee of the House of Commons on the Registration of Nurses, and Minutes of Evidence. (London, Eyre and Spottiswoode. One shilling.)

### Lectures upon the Nursing of Infectious Diseases.

By F. J. Woollacott, M.A., M.D., B.S.Oxon, D.P.H., Senior Assistant Medical Officer, Park Hospital, Metropolitan Asylums Board, Hither Green.

### LECTURE VI.—THE COMPLICATIONS OF SCARLET FEVER (*continued*).

The two most characteristic complications of scarlet fever are inflammation of the joints and inflammation of the kidneys. The former of these is commonly called scarlatinal rheumatism. As a rule it begins quite early in the disease, towards the end of the first week, and sometimes before the rash has faded. It shows a special tendency to attack adults and older children. In character it closely resembles acute rheumatism or rheumatic fever of a rather mild type, the chief symptoms being pain and swelling of the joints. The pain is increased on movement. Any joint may be affected, but the wrists and fingers suffer most frequently. As in acute rheumatism, though less often, the inflammation may attack the heart, causing pericarditis, and occasionally valvular disease. This constitutes the chief danger. In young children the symptoms may be very slight, without joint swelling and with very little pain. The only sign may be that the child lies rather more quietly than is natural, and if moved cries out. When the nurse notices this she should report it, for however slight the symptoms may be, there is always a risk that the heart may be damaged.

The treatment is precisely the same as in acute rheumatism: rest, warm clothing, and a simple milk diet. It is a good plan to wrap the affected joints in cotton-wool. This will tend to restrain movement, and will to some extent ease the pain. If the heart be involved, prolonged rest in bed is essential.

Occasionally the joint-inflammation becomes more acute and suppuration may occur. This, however, is more common when the joint mischief begins comparatively late in the disease. The only remedy is to wash out and perhaps drain the joint.

Inflammation of the kidneys, or nephritis, is one of the later complications of scarlet fever, and usually begins at the end of the third week. It is characterised by the presence of blood and albumen in the urine, and its onset is attended with headache and perhaps vomiting and shivering. We frequently notice that a day or two before the blood appears the patient seems out of sorts, with perhaps a slight rise of temperature. Sore throat may be complained of, ear discharge may appear, or the glands of the neck may become swollen and tender. The appearance of these symptoms in the course of the third week should put us on our guard. The patient should be kept in bed, his bowels freely opened, and the urine should be saved each day for testing.

The changes in the urine will be readily understood if we consider the work the kidneys have to perform. It will be remembered that their function is to remove waste matters and surplus water from the blood. They may, in fact, be regarded as a pair of living filters, which allow only impurities to pass through. This is their action in health. When attacked by disease they behave very much like filters when they get out of order. They may become blocked so that very little is able to pass through, or, on the other hand, they may permit the passage of substances which it is their duty to keep back. Both these results are seen in acute nephritis. The kidney secretion is diminished in quantity, and contains blood corpuscles and albumen. The urine may vary considerably in appearance. If there be much blood it is almost black, but more commonly it is red or smoky, while the deposit is of a chocolate colour. These peculiarities are very characteristic, but tests should always be applied. The simplest are the following: Take a little urine in a test tube and add a few drops of tincture of guaiacum. A milky fluid results. To this add some ozonic ether. When blood is present a blue colour appears at the line of junction of the two fluids. To test for albumen the best way is to boil a little urine in a test tube. Should it become turbid or throw down a deposit add a few drops of acetic acid. If the turbidity remains unchanged, then we are certain that it is due to albumen. The quantity of urine passed should be carefully noted, and the amount for the 24 hours entered on the chart.

In the treatment of the case the following are the main points. The patient must be kept warm in bed and carefully protected from draughts. A long flannel nightdress should be worn, the sheets should be removed and he should lie between blankets. Our object is to increase the action of the skin, so as to compensate in some degree for the failure of the kidneys to do their work. In severe cases, when the flow of urine is much diminished, it may be necessary to promote perspiration by baths of warm water or hot air. As a rule they should last about 20 minutes, and afterwards the patient should be wrapped in warm blankets and take a hot drink. A free action of the bowels should be secured daily. The food should be light and simple, and if the case be severe, only liquids should be given. The patient must be encouraged to drink freely, in the hope of flushing the kidneys and removing the inflammatory products which are choking them up. Milk is our sheet anchor, but lemonade, weak tea, barley, or plain water are all useful. Beef-tea, or any animal broth or extract must never be given. As the urine becomes free from blood and albumen the diet is gradually increased, and the other restrictions cautiously removed. Convalescence is as a rule slow.

At times the case presents symptoms of a graver character. Thus vomiting may be very troublesome and rigors may make their appearance. A blotchy rash is occasionally present, best marked on the limbs and back. To check the vomiting the milk must be given in small quantities, and it is a good plan to peptonise it. When a rigor begins give the patient a hot drink, put hot bottles in his bed, and wrap him up well in his blankets. In the worst cases, in which the urine is greatly diminished or even entirely suppressed, symptoms of uræmia may supervene. The most characteristic of these are convulsions and drowsiness deepening into coma. The former are usually ushered in by slight twitchings of the face or limbs, and if these are treated in time the general convulsion may be prevented. Therefore, as soon as they are noticed, report at once to the doctor. Excessive drowsiness, too, should always be reported. The usual treatment of these conditions is the administration of an aperient, or an enema if vomiting be present, and a hot bath. For the convulsions chloroform is frequently used.

In other cases the heart or lungs may be attacked by various inflammatory affections, the chief symptoms being pain in the chest and difficult or rapid respiration.

The great majority of patients suffering from scarlatinal nephritis make a good recovery and the urine becomes quite normal again. On the other hand death may occur, or the disease may become chronic. This latter result is most common when the case has been neglected in the early stages, as, for instance, when it has not been recognised that the patient is suffering from scarlet fever until the actual onset of nephritis. In these cases too well marked dropsy is frequent, whereas in patients properly treated it is absent, or present only to a very slight extent.

Apart from acute nephritis, albuminuria may appear at any period of the disease. It is customary, therefore, to test the urine at least every other day.

During scarlet fever, and especially in the later stages, hospital treated patients show a tendency to develop diphtheria. The main symptoms are sore throat, swollen glands in the neck, nasal discharge, or croup, with rise of temperature. The onset of any of these should be reported at the next visit of the doctor.

As a consequence of the attack of scarlet fever various ill effects may be left. Anæmia and general weakness may long persist, especially after rheumatism or nephritis, and, as we have seen, the heart and kidneys may be permanently damaged. Discharge from the ears, too, may continue for many months, and occasionally deafness follows. Fortunately the great bulk of the patients, if skilfully treated, make a recovery complete in every way.

Scarlet fever continues to be infectious, as a rule, till the peeling stage is completed. In uncomplicated cases the time is from six to eight weeks usually. It is the general practice to keep the patient isolated until the peeling has finished and until any discharges from the ear and nose have cleared up. The nasal discharge especially is very liable to contain the virus of the disease, and so may act as a source of infection. The scalp, too, must not be overlooked, and throughout the illness and at its close it should be kept free from scurf. During the peeling stage it is a common plan, if the patient be isolated at home, to oil the skin daily after bathing, the object being to prevent the little scraps of loose skin from becoming detached and blown into the house. In hospitals this is not found necessary.

### Beyond the Seas: Nursing in the Australian Bush.

Australians, to get the full benefit of their delicious climate, often camp out during the summer months, either in tents or wooden shanties. In this case a mother and father determined to sacrifice their comfort for their three children's pleasure, and chartered a disused boat club-house by the river, about half a dozen miles from the city by Bush or ferry. It was a beautiful spot near a bridge and a jetty. There was no other habitation within miles, except a small public-house and a few camps rigged up by young men. It was here that the youngest member of the family fell ill with typhoid fever. The doctor, whom the father brought down by boat to see the boy, advised instant removal to a city hospital. But no! the mother must nurse him; so the doctor could only leave instructions, and be reported to every day by the father, as it was too far to call regularly. Probably in England he would have washed his hands of the case; but this was in the Bush, where you always have to trust to luck, and take things as they come. Of course, the case did badly. The mother was practically inexperienced, and the heat of the weather grew intensely. By-and-bye the little girl went down, and afterwards the mother. The doctor sent down a nurse. She was a German, and ran away in a couple of days. It was at this time, while lolling in a hammock on our front verandah, overlooking the river, and longing to be once more in a hospital, that I noticed a boat come round the point, and push towards the house. A man got out and climbed the garden. He looked burdened with sorrow, and approaching, asked nervously if it were true that one of us was a nurse. On reassuring him, he told me the story of the typhoid case at the camp and finally implored me to accompany him back, to look after his wife and family, until he could get another nurse. Handing him over to my mother I tumbled into uniform. He borrowed a lot of things, and we went off together. The elder boy was in the boat, and they rowed up stream in less than an hour. The club was a two-storied wooden shanty, the lower floor consisting of one large and two very small rooms, and a rough kitchen containing a camp oven with a tin funnel chimney, which sent its fumes straight to the spot where the sick children lay, and nearly drove them mad, when they reached the hungry stage, with the desire to devour what they could smell being cooked. The upper room was not enclosed, a rail only running round it, except at one end where they had slung hessian to form an oblong tent. This floor was reached by a kind of ladder staircase in the open, the whole being shaded by a corrugated iron roof. There was one miserable servant girl, with a child running at her heels, who did the washing, and looked after the father and schoolboy when they were home from the city.

### The Patients.

All this was explained to me as we ploughed through the sand and climbed the staircase. On the open landing stood a home-made deal table covered with paper, on which were the drinks and cookery for the invalids, and by it a three-sided deal box containing an oil lamp with which I afterwards did their cooking, the broth being made by standing a pickle jar in a billy of water over it. Lifting up the hessian, we stood before the invalids. The impromptu hospital was delightfully simple in its arrangements. It just held a chest of drawers, a chair, and the four beds which lined the sides at each end, and the back. The mother was at one end, scarcely ill yet; the little boy at the other, insensible, delirious, fearfully bad; the little girl near her mother, a gentle, quiet, little creature, with no bad symptoms except a persistently high temperature. The mother began to fret and pour out her woes directly. The other nurse had been so cruel, had made her get up to have her bed made; had never sponged her, never washed the feeder, giving them all drinks out of the same, forgot the medicine, never gave stimulant, was rough with the children, and, worst of all, had given the boy toast and butter because he screamed for it. That is what they had quarrelled about. I asked if she had left a report? she had not. A chart? she had not. I set to work to fix the patients all up. It took me till dinner-time, for they had been so neglected. The boy's temperature was up to 106°. I was not surprised after the toast episode, though I had found all but one bite under the blankets. The girl's was 104°, and the mother's 99°, low enough for her to be able to keep an eye on the children when I went to meals. She had a bell at hand to enable her to summon help if necessary. We had meals in the big room when the menkind were at home. They slept in one little room, while the servant and her baby occupied the other. My bed was the fourth in the camp; it was a wire stretcher without a mattress, but it was cool, and prevented my going to sleep too heavily. The doctor came down the next day and left me instructions as to how to proceed, and I think we understood each other. Days passed, and I noted that the family said nothing about obtaining another nurse, but as I got interested in the cases, I should not have liked to leave them. The little boy became fearfully worse, especially at midday. His temperature came down at night, or he never would have pulled through, for I could not have sponged or done much for him then, when it was cold. I could only have my bed close and wake up when he wanted anything. An anxious patient makes one wonderfully alert in that way, and I managed to be up regularly for 4-hour drinks all through the three months I

### BEYOND THE SEAS: NURSING IN THE AUSTRALIAN BUSH—*Continued.*

was with them. The little girl was a very slow, persistently bad, but steady patient, while the boy went up and down like the weather. He developed meningitis, which nearly proved fatal. This made him scream and swear, for he had been out fishing with the soldiers and had picked up some of their worst words. These he would bawl at his mother when she coughed, at his sister when I paid her any attention, and at the flies when they worried him. But one day, when a clergyman came to see them, he sang hymns from morning till night till the others went into hysterics.

### An Attack of Pneumonia.

The worst of the whole business was having to leave them while I attended to the sanitary arrangements. My orders were to make a bonfire and burn everything, which was easier said than done, with a blazing sun 104 degrees in the shade. A mushroom hat took the place of a cap, and I wore hard boots to plough through the sand. I would be down making my wet fire burn, perhaps for the twentieth time that day, when the boy would begin like this: "If you don't shut up, mother, I'll get out of bed, I will. I'm going to do it," followed by the most dreadful swearing. Flying to the foot of the ladder I would hear the mother's soothing voice. Then, after a time: "Well, if you will shut up I'll keep quiet." About this time the night winds got bitterly cold and the mother got pneumonia. Never shall I forget the day she developed it. She was tired, cross, and hot, with cold feet. A beer bottle of hot water warmed them; but, alas! it burst and saturated the mattress. Over this she grew hysterical and flatly refused to go on my wire bed. I was in despair: there was nowhere else to put her. Then Billy came to the rescue. "Put Elsie on the end of my bed and mother in hers." He was watching us, in one of his intervals of consciousness, with his bright eyes. There was nothing else to do. Elsie was like a feather to move, and very good. She lay for the next hour or so at the other end of his bed, enjoying the novelty of the situation. The mother in reverse was heavy and awkward to move, being hopelessly frightened. And she was fretful, miserable, and uncomfortable, so that her temperature ran up as high as Billy's. I thought that mattress would never dry, though in truth it was wonderfully quick in the sun. Next morning the mother had grown alarmingly ill, and I sent an urgent message to the doctor to come down during the day. He did not come, nor did he send a message; in fact, as was often the case, no one came near us all day. When at last the father came home, he said, casually, "I saw the doctor to-day, and gave him your message. He said he would run down to-morrow night."

"But your wife is very bad," I ejaculated.

"Is she troublesome?" he said. He had hit the right nail on the head, but I could not answer him truthfully. I was annoyed, anxious, and overstrung; and I said, as quietly as I could: "If you will take charge of the patients after dinner, I will go out for a spell." He made no objection to this, so the schoolboy rowed me home; and that pull over the quiet water braced me up to start again. Next day I told the father he must send the doctor down early, and desired the servant to meet every ferry. He did not come. The mother was very sick, and would lean up on her elbow. She had a bad pain in her side, and fretted for linseed poultices. These I refused, substituting home-made mustard leaves, at which she grew very annoyed, and said: "You may understand typhoid, Nurse, but you certainly do not understand 'lungs.' You should do this or that." Then she continued: "I think I am going to die, and I know you won't tell me if I am." It was a most miserable day altogether. The mother's fretfulness reacted on the little girl so that she, too, got querulous. This irritated the boy, and he made a great fuss in his own way, swearing at his mother till she sobbed. All the temperatures went up, for the day was scorchingly hot, and I had to "pack" the children constantly. When it grew cooler, they quieted down tired out, and by the time the evening ferry was expected, they were all asleep. Slipping on my hat, I ran down to meet it. As I expected, there was no doctor with the father, who looked scared to death when he saw me. I only said: "The patients are all right so far. I must see the doctor, so I am going up. Everything is ready—medicine and drinks; but don't wake them for anything." With that I jumped on the receding steamer.

### Interviewing the Doctor.

It was so cool and fresh on the boat, as it glided softly through the eau-de-nil water, with the sweet sea breeze blowing up through the gathering twilight, that I felt as though the day had not been real, but rather a bad nightmare. My head though told a different tale; I could scarcely hold it up. On reaching the doctor's, I behaved like a coward, being nearly on the verge of hysterics. I walked in and said: "I have come to say I can't stay at that place on the river any longer; it is more than human nature can stand. Day and night, night and day. The boy dying, the girl crying, the mother sighing. She's lost all faith in me since I won't linseed-poultice her, and you've not been down or even sent me a message." The old doctor took my elbow, pushed me into a chair, and turned the light full on my face. "How long have you felt like this?" he asked.

"Since yesterday morning, when I sent for you."

"I had no message yesterday—to-day."

"Then you do not know that your lady patient has developed pneumonia?"

"No, I sent some liniment down to-night for a pain in her chest. I had no message to say I need come."

"But you will. I do not know if she is bad or not."

"Do you think you have taken this fever?" suddenly.

"The fever only of impatience and excitement. I am on the point of screaming."

"Don't do that. Take this thermometer. Ah, that's all right. I will mix you a powder. Now tell me all about it." I did, then he said: "I wonder how you have stood it all the time. When the boy got worse I tried to get another nurse to go and help you, but could not, so I had to let things go, simply watching you. And now that the crisis has come, I don't see what we are to do."

"Couldn't you manage to get the mother to hospital? I could go on nursing the children—should like to."

"I could, and would certainly, but she won't come. I tried to get them up from the start. As far as the case is concerned, the risk of moving is less than the risk of pneumonia in such a place, with the hot days and the cold nights. That is why you were right about the poultices."

"Do let us try to get her here, for I really cannot bear the responsibility longer."

He was silent for a long time. Then he said brightly: "This is what I have decided to do. Write to the husband and tell him that I advise removal, why, and how to do it. Then say that if they do not do it the responsibility will rest with them. I will continue to do my share towards their recovery, and you—will you continue to do your best? though their ingratitude hurts you, I can understand. You must know that you are their mainstay, that they could not do without you. Besides, you are doing splendidly from a professional point of view. Will you not stick to it?"

Being a soldier's daughter there was nothing else to do, and so he wrote the letter.

"If they don't come to hospital, nurse," he remarked, before I left to catch the last ferry home, "You had better get them down into that big room below."

"Down that ladder? A typhoid? Oh, doctor!"

"You'll do it," he chuckled. "You'll do it."

We did it; but it nearly broke the husband's back—and mine.

(*To be concluded.*)

### Examination Questions for Nurses.

Opening of the New Session.

Almost all nurses are now back from their well-earned holidays with, we hope, renewed health and strength, and fresh energy to cope with the trials and difficulties of their calling. Let me beg those who will compete in the montbly examinations this year, to bring their best efforts to the work. What is worth doing at all is worth doing well. When I see careless writing, to say nothing of faulty matter, blotted pages, sheets torn out of old ledgers, papers precariously tied up with thread, and no envelopes, I say, "Behold a slovenly nurse! one probably who will not wash medicine glasses, nor cleanse the spouts of feeding cups." Is it not true that a woman who is slovenly in trifles, will be so also in serious matters? But in truth there are no trifles in nursing.

I have had occasion before to warn competitors against the danger of intruding into the doctor's province, and I must beg for indulgence whilst I repeat that warning.

In these days, when nurses are so much more instructed than they were 10 or 15 years ago, it is sometimes a temptation to them to trust a little too much to themselves, instead of carefully and conscientiously carrying out the instructions of the medical man in attendance. Always remember that it is the sign of a first-class nurse, to obey the doctor's instructions even in the most minute particulars. Be careful too, not to intrude your opinions and theories on him. Your place is to state facts to him and leave him to base his own theories on them. If a nurse be intelligent and capable he will often ask for her opinion with regard to certain symptoms, an opinion which in his absence she alone can form—but she should on no account volunteer ideas and conjectures.

Once more let me ask for simplicity in the papers and a stern adherence to the matter under debate. Do not go off on branch railways, but stick firmly to the main line. Answer the question and nothing but the question, at the same time try to see that question in all its bearings, not from one side only. Above all things be practical—do not be taken up with showing off your erudition. Alas! that is a snare to many; their papers overflow with learned phrases—often incorrectly spelt—but practical knowledge is conspicuous by its absence. There is, however, a very decided improvement in the papers during the last six years, and it is pleasant to find that nurses abroad seem to value our efforts to bring them into working relations with ourselves.

Question for October.

If called upon to nurse a case of scarlet fever in a house where there were other inmates, what steps should you take for the prevention of the spread of infection?

Question to run concurrently with that for nurses abroad. This question has been asked not long ago, when the answers were not very satisfactory. It is, therefore, well to repeat it; moreover, it is our plan to set the same question for all nurses whether at home or abroad.

The Examiner.

Rules.

The competition is open to all. Answers must not exceed 500 words, and must be written on one side of the paper only. The pseudonym, as well as the proper name and address, must be written on the *same* paper, and *not on a separate sheet.* Papers may be sent in for fifteen days only from the day of the publication of the question. All illustrations strictly prohibited. Failure to comply with these rules will disqualify the candidate for competition. Prizes will be awarded for the best two answers. Papers to be sent to "The Editor," with "Examination" written on the left-hand corner of the envelope.

In addition to two prizes honourable mention cards will be awarded to those who have sent in exceptionally good papers.

N.B.—The decision of the Examiner is final, and no correspondence on the subject can be entertained.

Any competitor having gained three prizes within the current year shall be disqualified from taking another until 12 months shall have expired since the first prize was gained.

### Everybody's Opinion.

### THE TRAINING OF MIDWIVES.

The Secretary of the Association for Promoting the Training and Supply of Midwives, Dacre House, New Tothill Street, Westminster, writes: Kindly allow me to state that the pupils to whom you referred last week are the first trained under new conditions, and for whose services the committee invite application to this office. Heretofore, those trained under Miss Katherine Twining of Plaistow have gone direct from her to district nursing.

### HEXHAM GUARDIANS AND THEIR HEAD NURSE.

"B. O." writes:—Your informant is not correct in stating that the former head nurse of Hexham Workhouse was not hospital-trained. She had received her three years' training, also maternity training, and had ten years' nursing experience. She resigned her appointment when the Guardians wished to place the assistant nurse—a woman with no hospital training—on an equality with her.

[The question of the training of the head nurse was not mentioned by us, and our comment was that it was objectionable that she should be placed on an equality with untrained nurses.—Ed. The Hospital.]

### GOOD NURSING?

"Night Superintendent" writes: There is a point in nursing upon which I should like the opinion of others who, like myself, have finished their training and have had opportunities of noticing the "pros and cons" of the different ways of preparing cases prior to operation—I mean as to the thorough purging beforehand and its effect on the patient. We have all been taught in our lectures that it is indispensable to the welfare of the patient, that he should have a good night's rest with nothing to excite him before undergoing his operation, whether it be of a major or a minor nature. Now I know, only too well, this is very seldom effected. And why? Let me describe a case in point and the reason will be evident. Mrs. D. is admitted to our hospital on Tuesday, suffering from abdominal tumour. The visiting surgeon sees her on Wednesday and says he will operate the following day. An aperient is therefore given in the evening and Mrs. D. spends the first five or six hours vomiting, can keep nothing down, and so nourishment is out of the question. At 2 a.m. her condition is the same. At 3 o'clock an enema is administered; the vomiting ceases but patient is too upset to take anything for at least an hour, when we try her with a little tea, to pave the way for the milk we hope to get down later, but which she cannot manage yet. Fairly worn out she sleeps for two hours, and is then ready for the milk—her first nourishment since 8 p.m., and it is now 6 a.m. But her troubles are not yet over. There has as yet been no result from the castor oil of the preceding night, so a second enema must be administered, and after a cup of bovril at 7 a.m., the last food she can have before the anæsthetic, we at last leave her in peace, though not to sleep, for now the day's work begins in the ward and all is noise and bustle. This is only one of many similar cases I have seen, and the surgeons operate happily, under the delusion that the patients have been well prepared, and wonder why there are so many cases of collapse even in minor operations! Small wonder indeed when the patient, has put through a night like the above, with scarcely any rest or nourishment. No, there is both good and bad nursing, and, in my opinion, one based on long experience and special noting of the question in hand, the good nursing is that of the ward sister whose rule it is to administer an aperient during the morning hours of the day before the operation, which can then be repeated, if necessary, before tea, and a copious enema given the last thing before the ward settles down for the night, generally about 8 P.M. I can answer for this method being far more efficacious and at any rate securing a quiet night for the patient with opportunity for administering nourishment, such as is allowed. Of course in some cases special orders are given, but, as a rule, the doctors leave this part of the nursing to the common sense of the ward sister. If night nurses were more careful to report these bad nights, I feel sure the sisters would be only too glad to manage differently.

### QUALITY OF TRAINING.

The Honorary Secretary of the North Cambridgeshire Hospital, Wisbech, writes:—In your "Nursing Outlook" in The Hospital of September 17th some incorrect statements are made as to the North Cambridgeshire Hospital, Wisbech, which, as honorary secretary, I should be glad if you would rectify in your next issue. At Wisbech there is a very complete operating theatre, fitted up on the most modern and sanitary principles, and with the newest appliances. The operations undertaken by the honorary surgeons and other medical men would compare with those of the larger hospitals, and cases are not sent away, as ludicrously suggested, to the "nearest big town," but, on the contrary, eminent medical men from Cambridge and elsewhere find the operating theatre so convenient and well-fitted that they are able to undertake the most difficult and serious cases. There is a resident medical officer, matron, and an efficient staff of nurses, an out-patient department, and all the medical men of the town attend in rotation, choosing two of their number as honorary surgeons, who represent them on the committee. It is not correct that a premium is taken from probationers, and the certificate awarded to them when they leave always states the time that they have served in the hospital. Of course, the training of larger hospitals may, in some respects, give a wider experience, but there is not room for all those requiring to be taught, and in an institution like the one at Wisbech, so greatly misrepresented in The Hospital, a very good knowledge of nursing may be obtained in two or three years, which with extended experience elsewhere later, will equip a nurse efficiently for her duties.

The Matron of Porth Cottage Hospital writes: With reference to an article which appeared in The Hospital of September 10th on so-called training in cottage hospitals, I beg to make a few remarks. I myself spent six years in University College Hospital, one of the leading "medical schools" and recognised training schools for nurses, and like all students and nurses probably consider my own hospital as perfect in every way, and would be the last person to underrate the value of a three or four years' course in a large London hospital, or to wish to flood the nursing ranks with half-trained and very young women. At the same time, I must express a conviction that if a probationer rightly makes use of her time and opportunities during a course of twelve months in even a small cottage hospital, she can acquire a very good insight into the art of nursing critical and helpless cases, for a probationer in a small hospital is taught to do things which a fully-trained nurse would not be allowed to see in a medical training school. Our hospital is in the midst of a large colliery district where serious mining accidents are of frequent occurrence, and is purely for surgical and gynæcological cases. I enclose a copy of our last year's report which has just been printed, and beg to draw your attention to the fact that we do not send our worst cases to the nearest town, but we have honorary consulting surgeons upon our medical staff who are attached to a recognised hospital, and they most kindly arrange to come up and operate whenever their services are requisitioned. At the same time they show their confidence in our nursing staff, inasmuch as they leave their cases entirely in our hands without introducing their own special nurses. Our number consists of a nurse who has been engaged here now for fully three years, first as probationer and latterly as "charge," and two probationers, beside myself. You will also observe that it is not such a struggle with us, as with some, to pay our way. Our little hospital is well supported, as our receipts will show, and, thanks to the unflagging interest of both committees, is thoroughly looked after in every way.

### Appointments.

Bolton Infirmary.—Miss Kate Trow has been appointed sister. She was trained at Shoreditch Infirmary, where she has since been staff nurse.

City Hospital, Park Hill, Liverpool.—Miss M. H. Christie has been appointed matron. She was trained at Toxteth Infirmary, Liverpool, and has since been charge nurse and night superintendent at Prescot Infirmary, and assistant matron at the City Hospital, Park Hill, Liverpool.

Clapham Maternity Hospital.—Miss Lucy E. Ashby has been appointed charge nurse. She was trained at St. Bartholomew's Hospital, Rochester, and has since done both general and maternity nursing. She holds the L.O.S. certificate.

Cumberland Infirmary, Carlisle.—Miss C. Richardson has been appointed night superintendent, and Miss C. L. Lees sister. Miss Richardson was trained at the Bristol Royal Infirmary, and has since been sister in that institution and sister at Cumberland Infirmary. Miss Lees was trained at Brompton Consumption Hospital and Radcliffe Infirmary, Oxford. She has since been, temporarily, sister at the East London Hospital for Children, Shadwell; night superintendent at the North-Eastern Hospital, London; and night superintendent at St. Mary's Hospital, Plaistow.

Dr. Matthews' Private Hospital, Johannesburg.—Miss Elizabeth Lord has been appointed matron. She was trained at Guy's Hospital, London; she has also had fever training, and holds the L.O.S. certificate.

Grove Hospital, Tooting.—Miss Norah M. Redmond has been appointed charge nurse. She was trained at Shoreditch Infirmary, where she has since been staff nurse.

Hull Sanatorium.—Miss Lilian Amelia Parsons has been appointed sister. She was trained at Stapleton Infirmary, Bristol, the Royal Infirmary, Bristol, and the Dartford Isolation Hospital, Kent. She has since been charge nurse in the typhoid wards of the City Hospital, Liverpool; has done matron's duties at the Hertford Sanatorium, Alcester, Warwickshire, and at the Hastings Sanatorium; she has also taken night superintendent's duties at Leeds General Infirmary. For the past 10 years she has been engaged in private nursing.

Isolation Hospital, Crewe.—Miss Mary G. Currie has been appointed matron. She was trained at the City of Glasgow Fever Hospital, Belvidere, and the Western Infirmary, Glasgow, where she afterwards held the posts of night superintendent and ward sister. She has also been matron of the Infectious Diseases Hospital, Airdrie, and assistant matron, Gartloch Hospital for Mental Diseases, Gartcosh.

Johannesburg Hospital.—Miss Lily Pirt has been appointed sister. She was trained at the Royal Infirmary, Bradford.

London County Council Schools.—Miss Esther E. Clements has been appointed nurse. She was trained at Mile End Infirmary and has since been sister at Poplar and Stepney Sick Asylum, charge nurse at the Brook Hospital, ward sister and maternity sister at Shoreditch Infirmary. She holds the L.O.S. certificate.

Plaistow Fever Hospital.—Miss Augusta W. Hendry has been appointed staff nurse. She was trained at Shoreditch Infirmary where she has since been staff nurse.

### "The Hospital" Convalescent Fund.

The Hon. Secretary begs to acknowledge, with thanks, the receipt of 10s. from a donor who wishes to remain anonymous, and 2s. 6d. per the Travel Correspondent of The Hospital.

### Echoes from the Outside World.

### Movements of Royalty.

THE King concluded his visit to Glenquoich on Monday, and in the evening arrived at Balmoral Castle and was received by a guard of honour at Ballater Station. A motor-car was in waiting, and as his Majesty drove off in it to Balmoral, loud cheers were raised by the assembled crowd. Mr. Balfour, First Lord of the Treasury, has arrived at the Castle as Minister in attendance.

The Queen, who is still in Denmark, was present at a dinner given in her honour at Charlottenlund Castle on Friday night. On Monday her Majesty visited Copenhagen, and on Tuesday she was present at the ceremony of unveiling a monument to Count Bernstoff. She has been pleased to learn during the last week the gratification given by her presentation to the Royal Victoria Military Hospital, at Netley, of the Royal crimson felt seating, richly bordered, with which, at her instruction, the chapel has been fitted.

### Queen Alexandra's Homes at Wimbledon.

THIS week the central block of the Homes for Officers' Widows and Daughters at Queen Alexandra's Court, Wimbledon—close to St. Mary's Church and the Park—will be open for the reception of twelve families, twelve more being accommodated at the end of next week. The buildings, erected on ground three acres and a half in extent, are mainly the outcome of the Queen's gracious action in devoting £10,000, her Coronation gift, and £5,000 from her War Fund, to their establishment. When completed, the several other blocks will provide self-contained unfurnished apartments for thirty-six additional families. As Sir James Gildea, chairman of the Executive Committee of the Soldiers' and Sailors' Families' Association explained, all that the ladies in residence will have to pay for is the gas they consume, or the electric light they burn. To gain admittance they must neither be under 50 nor over 80, and they must have an assured income of not less than £40 and not more than £100 per annum. Each suite consists of a sitting-room—which is a fairly large apartment with parquet flooring to avoid the need of a carpet—two smaller bedrooms, and kitchen with cooking ranges, coal cupboard, and every convenience to dispense with unnecessary labour, for, naturally, the income of the inmates will not allow of a servant. The wives of the two men who attend to the gardens will keep the staircases clean. The buildings are in three stories, the first and second floors also possessing balconies, and so far they have been built free of debt, but £20,000 more is required for any further operations.

### Princess Christian in South Africa.

ON Saturday morning Princess Christian laid the foundation-stone of a home for the aged poor, planted a tree, and opened Princess Park. In the afternoon she opened a bazaar in the Zoological Gardens in aid of Church charitable funds. Addresses were read on behalf of the women of Pretoria, the Loyal Women's Guild, and the nurses, many of whom were presented to her Royal Highness. The route to the gardens was decorated, and was lined with thousands of spectators, who cheered the Princess warmly. The Johannesburg Cadet Corps, 250 strong, formed a portion of the troops in attendance. The Princess expressed her gratitude for the tender care which has been bestowed upon her son's grave, and, before leaving, she presented a signed portrait to the florist who attends to the grave, and thanked his children, who are in the habit of placing flowers upon it. On Monday she paid an official visit to Johannesburg, and on Tuesday laid the foundation-stone of a new wing of the Johannesburg Hospital.

### The War in the Far East.

IT seems doubtful whether there will be a renewal of serious fighting in the Far East at present. The Russians, who have been largely reinforced, are engaged in fortifying a position north of Mukden. The Tsar has resolved to form a second army in Manchuria, and has entrusted the command to General Gripenberg. His Majesty, in an autograph letter to this officer, says that the energy with which Japan is conducting the war, and the stubbornness and high war-like qualities displayed by the Japanese, compel him to make considerable additions to his forces at the front in order to attain a decisive success within the shortest possible time. According to advices received in St. Petersburg from Chifu, an assault made by the Japanese on Port Arthur last Friday after four days' bombardment was repulsed with heavy loss.

### Illness of Lady Curzon.

ON Friday the appointment of Lord Curzon to be Governor-General of India was gazetted, but on the previous Wednesday Lady Curzon became seriously ill, and all arrangements for the departure of their Excellencies were, of course, set aside. On Thursday Sir Thomas Barlow, Dr. Champneys, Dr. Watson Cheyne, C.B., and two local doctors were in attendance. Lady Curzon is suffering from peritonitis, and on Saturday an operation was successfully performed by Dr. Watson Cheyne. Lord Curzon remained constantly at his wife's bedside last week, and did not go outside the Castle until Monday. Mrs. Leiter, Lady Curzon's mother, and her daughter, on receipt of the news, travelled as rapidly as possible from Denver, and with great difficulty caught the Red Star liner *Vaderland* which sailed on Saturday for Europe. Their cabs appeared on the scene just as the whistle of the liner sounded. Sailors were standing ready to assist the travellers on board, and the instant they left the gangway the ship was got under way. The utmost concern is expressed in Simla at Lady Curzon's illness, and expressions of sympathy have reached the Viceroy from all parts of the world. The King has made frequent inquiries. An improvement in Lady Curzon's condition was reported early on Tuesday, the patient having had a little natural sleep, but in the evening she was worse. During Tuesday night she rallied.

### Pictures in Needlework.

IN these days when needlework is almost a neglected art, such beautiful specimens of embroidery as those now being exhibited at the Society of Artists, 52 New Bond Street, under the auspices of the Lyceum Club, are especially worth attention. The exhibition, which opened on Monday, consists of pictures in needlework, entirely the handiwork of Mrs. Florence Jessie Hösel. These are not copied from paintings, nor are they the result of sketches made by the artist herself, for Mrs. Hösel works entirely without designs, embroidering her pictures straight on to the material with great rapidity. In many cases, also, she dyes her own silks. One of the most striking examples is a wonderful table-top representing "The Four Seasons," the distinctive points of which are conveyed to the eye entirely by the difference in colour. In one scene the pale blue of the soft sky, flecked with white and the budding trees, betoken spring; in another the dull slate of the heavens and the winter nakedness of the snowy landscape, shows that the winter has returned. The water below reflects in each case the hue of the sky above. So elaborate is the detail in these pictures that the top took six months to complete, working 18 hours a day. "Midsummer Night's Dream," a mass of many-coloured butterflies in a meadow, and, No. 5, an autumn landscape, both deserve special notice. Many of Mrs. Hösel's designs for portières and for cushions are worked most effectively on linen, in one case 100 years old. This original exhibition should not be missed by all lovers of beauty, whether clever with their needle or otherwise.

### Notes and Queries.

**REGULATIONS.**

**The Editor is always willing to answer in this column, without any fee, all reasonable questions, as soon as possible.**

**But the following rules must be carefully observed:—**

**1. Every communication must be accompanied by the name and address of the writer.**

**2. The question must always bear upon nursing, directly or indirectly.**

**If an answer is required by letter a fee of half-a-crown must be enclosed with the note containing the inquiry.**

*Homes.*

I should be much obliged if you could tell me where I could obtain information about the homes for epileptics at Bushey.—*M. E. H.-B.*

Do you mean the Chalfont Colony Homes? If so, write to the Secretary of the National Society for the Employment of Epileptics, 12 Buckingham Street, Strand, W.C.

Can you kindly give me the names of homes where simple-minded women over 30 may be received on payment?—*L. I.*

Write to Secretary, The National Association for Promoting the Welfare of the Feeble-minded, 53 Victoria Street, S.W.

Can you tell me of a home where a man, aged 36, suffering from nervous debility could be received? His home has just been broken up by death, but his relatives could afford to pay 10s. or 12s. weekly for home. He is able to do for himself.—*Nurse Ellen.*

Write to St. Peter's Home and Sisterhood, Mortimer Road, Kilburn, N.W.

*Australia.*

(2) Will you kindly give me the addresses of nursing homes in Sydney and other towns in Australia? I am anxious to go out there this autumn.—*E. C.*

See "Burdett's Hospitals and Charities," in which a list appears.

*Nursing Homes.*

(3) Can you give me any information as to nursing homes in Birmingham?—*N. F.*

The Nurses' Co-operation and Nursing Home, 23 Francis Road, Edgbaston, Birmingham.

*Cards.*

(4) Will you kindly tell me the correct way for a certificated and registered midwife to have her cards printed. Should she put "Miss" or "Nurse"? Should the qualifications underneath be Cert. Obst. Soc. Lon., as well as Reg. Midwife? Or one without the other?—*Norman.*

Either "Miss" or "Nurse" is correct, but the latter is preferable. Both qualifications should be given.

*Advertising Medium.*

(5) Will you tell me the best papers in which to advertise for mental or weak-minded cases? I have a good home and, being a Guy's nurse and also a mental nurse, I should like to take patients, especially children.

One of the leading daily papers or the medical papers. It is possible that the National Association for Promoting the welfare of the Feeble-minded, 53 Victoria Street, S.W., might like to know of your home.

*Snoring.*

(6) I am a maternity nurse and a year ago I had influenza which caused me to snore in my sleep. It is so bad that I am afraid to go to sleep when on a case for fear of disturbing my patient. Will you kindly advise?—*M. R.*

Consult a medical man.

**Handbooks for Nurses.**

"How to Become a Nurse: The Nursing Profession." 2s. net; 2s. 4d. post free.

"The Nurses' Dictionary." By Honnor Morten. 2s. post free.

"A Handbook for Nurses." By J. K. Watson, M.D. 5s. net; 5s. 4d. post free.

"A Practical Guide to Surgical Bandaging and Dressings." By W. Johnson Smith, F.R.C.S. 2s. post free.

"The Art of Massage." By A. Creighton Hale. 6s. post free.

"Preparation for Operation in Private Houses." By Stanmore Bishop, F.R.C.S. 6d. post free.

The above works are obtainable of all booksellers, or direct from The Scientific Press, Limited, 28 & 29 Southampton Street, Strand, London, W.C.

### For Reading to the Sick.

ST. MICHAEL'S AND ALL ANGELS' DAY.

Gracious Lord, who watchest o'er me,
Give me love that knows no sleep;
Lest I fail, in ought before Thee,
Give Thine angels charge to keep.

Beauty, Lord, that speaks Thy glory,
Is on man, on sky, on sea;
May I, reverent read that story
Seeing as Thine angels see.

Blessed angels! whom to render
Help to me, my Saviour sends,
May I live, through that dear Sender,
Worthy of such heavenly friends.

From *Treasury of Meditation.*

The Holy Angels watched over, guarded, cared for our Blessed Lord in His human life. They comforted Him in His temptation. One strengthened Him in His agony in the Garden of Gethsemane. They worshipped Him. The Holy angels, we are told in Holy Scripture, are real living beings. They are in vast numbers. They love God with pure love. They are established in Grace. Let us think of them as sympathising friends. How they sympathised with all the work of redemption, above all, with the Redeemer Himself. Think how, in His darkest hour in Gethsemane it was a Holy Angel who felt His weakness and misery, and came, though His beloved disciples slept, and "strengthened" Him. On the morning of the Resurrection they were there. Full of sympathy, and thought, and devotion. They are drawn to the human race, for they were drawn to Him who is our Elder Brother.

Think how deeply they sympathise with us all. The Angel felt for Hagar in her fear, and desolation, and suffering, about her child in the desert. The very light of their admiration seemed reflected on the Martyr Stephen, in his last great defence. With love and wonder they watched the sufferings and courage of the apostles. (1. Cor. iv. 9.)

God works for us by means of His Holy Angels. We must remember that we have "come to Mount Zion, and to an innumerable company of angels." We have, if we will, the holy fellowship of these Blessed ones. What tender care and loving providence this shows for us. We feel less awestruck and less lonely if we realise that we are one of a great company. If we meditate upon our nearness to that "innumerable company" of glorious and majestic beings, and remember that they love goodness and are keenly interested in our spiritual welfare, that they rejoice in our repentance, and sorrow over us when we fail, life becomes a larger thing, when we realise that we are objects of interest to the holy, the beautiful, the blessed. O beautiful unseen world, peopled with the pure, the holy! What a "Mount Zion" God has called us to. He has made us companions of this "innumerable company." Let us live accordingly.

O Blessed Jesus, who did Thyself teach us of the sympathy and love of the Holy Angels, and when on earth, didst allow the sympathy and tenderness of these, Thy pure creatures, to comfort Thee, give us grace to realise more the great gift Thou has given us in their near presence and loving care, and to live more worthy of their holy fellowship, for Thy mercy's sake.—*Canon Knox Little.*

